# Fighting an old disease with next-generation sequencing

**DOI:** 10.7554/eLife.06782

**Published:** 2015-03-03

**Authors:** Anzaan Dippenaar, Robin M Warren

**Affiliations:** DST/NRF Centre of Excellence for Biomedical Tuberculosis Research, SAMRC Centre for TB Research and the Division of Molecular Biology and Human Genetics, Stellenbosch University, Stellenbosch, South Africa; DST/NRF Centre of Excellence for Biomedical Tuberculosis Research, SAMRC Centre for TB Research and the Division of Molecular Biology and Human Genetics, Stellenbosch University, Stellenbosch, South Africarw1@sun.ac.za

**Keywords:** tuberculosis, transmission, Malawi, other

## Abstract

Whole genome sequencing has revealed that most cases of tuberculosis in a high-incidence setting in Malawi were caused by just one lineage of the bacterium responsible for the disease.

**Related research article** Guerra-Assunção JA, Crampin CA, Houben RMGJ, Mzembe T, Mallard K, Coll F, Khan P, Banda L, Chiwya A, Pereira RPA, McNerney R, Fine PEM, Parkhill J, Clark TG. Glynn JR. 2015. Large-scale whole genome sequencing of *M. tuberculosis* provides insights into transmission in a high prevalence area. *eLife* **4**:e05166. doi: 10.7554/eLife.05166

**Image** The majority of tuberculosis cases in the Karonga District of Malawi between 1995 and 2010 were caused by one lineage of the bacterium *M. tuberculosis* (shown in red)

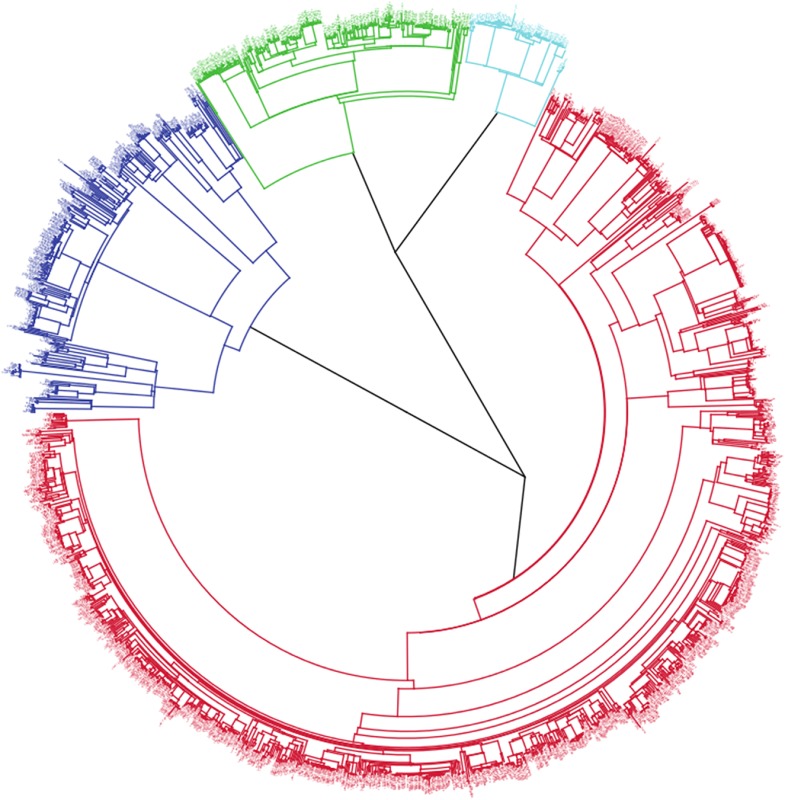

*Mycobacterium tuberculosis* is the causative agent of tuberculosis, a disease that is a major threat to human health worldwide. It is estimated that approximately 9 million people were diagnosed with tuberculosis during 2013, and that 1.5 million died from the disease. The global tuberculosis epidemic is being driven by co-infection with HIV and by the emergence and spread of drug-resistant strains of *M. tuberculosis*. The World Health Organization has reported that 3.5% of new tuberculosis patients, and 20.5% of patients who had been treated before, had multidrug-resistant forms of the disease in 2013. The diagnosis and treatment of drug-resistant tuberculosis is clearly a major global health challenge (World Health Organization, 2014).

The enormity of the tuberculosis epidemic has created a desperate need to develop methods to monitor the dynamics of the disease. In the early 1990s, the discovery of repetitive elements in the genome of *M. tuberculosis* laid the foundation for the development of the science of molecular epidemiology (van Embden et al., 1993). These methods have shown that in situations where tuberculosis is common, epidemics are driven by the transmission of the bacteria between individuals. However, in low incidence settings, epidemics are driven by the ‘reactivation’ of bacteria that have been lying dormant in individuals since an earlier infection. It is also known that recurrent disease—when the symptoms reappear after a patient has apparently been cured—can occur through a second infection event, and that drug resistance is spread by transmission (Mathema et al., 2006).

Traditional methods to identify strains of *M. tuberculosis* rely on the analysis of small windows of the genome, and it has been assumed that the DNA sequences in these windows are variable enough to allow researchers to separate strains of *M. tuberculosis* that are evolutionarily close or distant. However, the true complexity of disease dynamics cannot be resolved by tracking strains using a small section of the genome. The development of next-generation sequencing platforms has made it possible to view the complete genetic information of the bacteria, which should improve the accuracy of efforts to monitor strains of *M. tuberculosis* as they move through space and time (Roetzer et al., 2013). Rapid whole genome sequencing promises to be the ultimate tool for epidemiological investigations, diagnosis, and for testing whether strains of bacteria are susceptible to particular drugs.

Now, in *e*Life, Judith Glynn of the London School of Hygiene and Tropical Medicine (LSHTM) and co-workers—with Guerra-Assunção as first author—report how a long-term large-scale whole genome sequencing strategy has been used to decipher the tuberculosis epidemic in a high prevalence setting with multiple sources of infection (Guerra-Assunção et al., 2015). They analysed the whole genome sequences of 1687 *M. tuberculosis* samples (isolates) collected from patients in the Karonga District of Malawi over a period of 15 years. This represents 72% of the total number of confirmed tuberculosis cases during that time. The various strains of *M. tuberculosis* can be grouped into seven ‘lineages’ that each contain bacteria descending from a common ancestor. Guerra-Assunção et al. found that the epidemic was largely driven by members of one lineage, which implies that either this lineage arrived in the area earlier than the others, or that the members of this lineage were more successful.

The genome of *M. tuberculosis* consists of ∼4.4 million bases and is generally believed to be relatively stable (Jagielski et al., 2014). To identify isolates that were directly related in a transmission network (i.e., recently transmitted from one patient to the next), Guerra-Assunção et al. used a cut-off point of up to ten differences in single nucleotide polymorphisms between the genomes of the isolates. Next, they developed a clustering formula to group together directly related isolates. Using this formula in combination with network-analysis (where isolates are linked according to genome sequence similarity), they found that strains from certain lineages were more likely to be transmitted between patients than others. This suggests that there are differences in the abilities of bacteria in the different lineages to cause disease. In this high-incidence setting, 66% of identified cases clustered together, of which 38% of the patients had evidence of recent infection, implying ongoing transmission of the bacteria. This indicates that reactivation of previous infection was the primary driving force behind this epidemic.

Glynn, Guerra-Assunção and co-workers—who are based at the LSHTM, the Karonga Prevention Study in Malawi and the Wellcome Trust Sanger Institute—also showed that the proportion of tuberculosis cases due to reactivation increased over the duration of the 15 year study, as demonstrated by a marked decrease in transmission between 1999–2001 (45%) and 2008–2010 (30%). Guerra-Assunção et al. suggest that this decrease is due to the implementation of antiretroviral therapy and isoniazid preventative therapy in Karonga. However, this is counter-intuitive because both treatments should protect against reactivation, thereby raising an important question as to how reactivation may work in this context. Significantly, this study shows that the tuberculosis control program in Karonga has reduced transmission of the bacteria. It also demonstrates that whole genome sequencing can provide new insights into tuberculosis epidemics, which could be used to advise and fine tune control programs.

Despite the advantages of whole genome sequencing, it is important to acknowledge the complexity of the technology and data analysis. This questions how useful it could be in high-incidence settings where tens of thousands of cases are diagnosed annually. Furthermore, the current technology is restricted to clinical isolates that need to undergo a lengthy culturing and DNA extraction process, which prevents its use as a real-time monitoring tool. Additionally, whole genome sequencing is labor intensive and financially demanding, although costs have decreased significantly over the last decade. Regardless of these challenges, this technology has the potential to immediately revolutionise drug susceptibility testing by identifying the complete repertoire of mutations in target genes that confer drug resistance (Steiner et al., 2014). Application of this technology would decrease diagnostic delay, thereby reducing transmission, morbidity and mortality and, at the same time, improving treatment outcome.
